# *Scolopendra subspinipes mutilans* attenuates neuroinflammation in symptomatic hSOD1^G93A^ mice

**DOI:** 10.1186/1742-2094-10-131

**Published:** 2013-10-29

**Authors:** MuDan Cai, Sun-Mi Choi, Bong Keun Song, Ilhong Son, Sungchul Kim, Eun Jin Yang

**Affiliations:** 1 Department of Medial Research, Korea Institute of Oriental Medicine, 483 Expo-ro, Yuseong-gu, Daejeon 305-811, Republic of Korea; 2 Department of Internal Medicine, Wonkwang University of Medical School, Iksan, Republic of Korea; 3 Department of Neurology, Inam Neuroscience Research Center, Sanbon Medical Center, College of Medicine, Wonkwang University, Iksan, Republic of Korea; 4 Department of Acupuncture & Moxibustion, Wonkwang University Gwangju Oriental Hospital, 543-8, Juweol1-Dong, Nam-Gu, Gwangju 503-310, Republic of Korea

**Keywords:** Amyotrophic lateral sclerosis, *Scolopendra subspinipes mutilans*, Neuroinflammation

## Abstract

**Background:**

Amyotrophic lateral sclerosis (ALS) is a progressive, adult-onset neurodegenerative disorder characterized by selective motor neuron death in the spinal cord, brainstem, and motor cortex. Neuroinflammation is one of several pathological causes of degenerating motor neurons and is induced by activated microglial cells and astrocytes in ALS.

*Scolopendra subspinipes mutilans* (SSM) is utilized in traditional Chinese and Korean medicine for the treatment of a variety of diseases, such as cancer, apoplexy, and epilepsy. However, the mechanisms underlying the effects of SSM are currently unclear, even though SSM increases immune and antibiotic activity.

**Methods:**

To determine the effects of SSM on symptomatic hSOD1^G93A^ transgenic mice, SSM (2.5 μℓ/g) was injected bilaterally at the Zusanli (ST36) acupoint three times per week for two weeks. The effects of SSM treatment on anti-neuroinflammation in the brainstem and spinal cord of hSOD1^G93A^ mice were assessed via Nissl and Fluoro-Jade B (FJB) staining, and immunohistochemistry using Iba-1, CD14, HO1, and NQO1 proteins was evaluated by Western blotting.

**Results:**

In this study, we investigated whether SSM affects neuroinflammation in the spinal cord of symptomatic hSOD1^G93A^ transgenic mice. We found that SSM treatment attenuated the loss of motor neurons and reduced the activation of microglial cells and astrocytes. Furthermore, we demonstrated that SSM administration in this animal model of ALS suppressed oxidative stress in the brainstem and spinal cord by 1.6- and 1.8-fold, respectively.

**Conclusions:**

Our findings suggest that SSM, which has previously been used in complementary and alternative medicine (CAM), might also be considered as an anti-neuroinflammatory therapy for neurodegenerative diseases.

## Background

The neurological disorder amyotrophic lateral sclerosis (ALS) is characterized by selective motor neuron death in the brainstem, motor cortex, and spinal cord. Familial ALS (fALS) is caused by genetic mutations in several genes, including Cu/Zn superoxide dismutase (SOD1), alsin, senataxin, fused in sarcoma (FUS), vesicle-associated membrane protein-associated protein B (VAPB), TAR DNA-binding protein (TARDBP), and dynactin 1 (DCTN1) [1]. In addition, sporadic ALS (sALS), which accounts for the majority of ALS cases, is characterized by a toxic gain-of-function [1]. Mutations in SOD1 are responsible for 10-20% of fALS cases and have been used to generate an ALS-like animal model [2]. Mice expressing human SOD1 (hSOD1) recapitulate the paralysis observed in ALS patients. Although a number of pathogenic mechanisms underlying ALS have been reported, including oxidative stress, glutamate excitotoxicity, mitochondrial dysfunction, and protein aggregation [3], the overall disease etiology remains unclear.

Neuroinflammation is a common pathological process involved in many neurodegenerative diseases, including Alzheimer’s disease (AD), Parkinson’s disease (PD), and ALS [4]. Under physiological conditions, the inflammatory processes that occur in microglial cells promote innate immunity. However, under conditions of uncontrolled inflammatory stimulation, such as abnormal protein accumulation or stress, activated microglial cells and astrocytes increase the production of neurotoxic factors that induce neurodegenerative pathology. Several studies have demonstrated that the neuroinflammation caused by activated microglial cells is the hallmark pathological feature of ALS in animals and human patients [5-7]. Therefore, research has targeted therapeutic interventions that can ameliorate the effects of neuroinflammation to reduce motor neuron loss and disease severity in animal models of ALS.

The centipede, *Scolopendra subspinipes mutilans* (SSM), is utilized in traditional Chinese and Korean medicine for the treatment of a variety of diseases, such as cancer, stroke-induced hemiplegia, apoplexy, and epilepsy [8,9]. Several studies have demonstrated that water-soluble SSM extracts decrease tumors and increase immune activity in tumor-bearing mice [10-12]. Kim et al. reported that SSM treatment produces a significant increase in antibiotic activity against infections of the lung and intestines [13]. However, to date, the role of SSM therapy for neurodegenerative diseases has not been investigated. Our study assessed the effects of SSM treatment at the ST36 acupoint in hSOD1^G93A^ transgenic mice, an animal model of ALS.

We found that SSM treatment attenuated the loss of motor neurons and reduced neuroinflammation in the brainstem and spinal cord of symptomatic hSOD1^G93A^ transgenic mice, in addition to reducing oxidative stress in these structures. Based on these findings, we suggest that the administration of SSM may be helpful in reducing the severity of inflammation in neurodegenerative diseases.

## Materials and methods

### Materials

SSM were purchased from the Korean Pharmacoacupuncture Institute (Seoul, Korea). Cresyl violet acetate was obtained from Sigma (St. Louis, MO) and diluted with saline. Fluoro-Jade B was purchased from Chemicon (Temecula, CA, USA). An avidin-biotin peroxidase complex (ABC) kit, 3, 3′-diaminobenzidine tetrahydrochloride (DAB), and mounting medium for performing fluorescence analysis with DAPI were procured from Vector Laboratories (Burlingame, CA, USA). Alexa Fluor 488 goat anti-rabbit IgG (H + L) was purchased from Invitrogen (Carlsbad, CA, USA). Bovine serum albumin (BSA) was procured from Gendepot (Barker, TX, USA). The primary antibodies employed for Western blotting and immunohistochemistry were as follows: anti-Iba-1 (diluted 1:1,000, Wako, Japan), anti-GFAP (diluted 1:3,000, Millipore Corp., MA, USA), anti-MAP2 (diluted 1:500, Millipore Corp., MA, USA), anti-HO1 (diluted 1:1,000, Abcam, MA, USA), anti-NQO1 (diluted 1:1,000, Santa Cruz Biotechnology, CA, USA), anti-human SOD1 (diluted 1:2,000, Calbiochem, CA, USA), and anti-CD14 (diluted 1:500, BD Biosciences, CA, USA). Anti-α-tubulin (diluted 1:5,000, Abcam, MA, USA) was used as a loading control.

### Animals

Hemizygous hSOD1^G93A^ transgenic (Tg) mice were purchased from the Jackson Laboratory (Bar Harbor, ME, USA) and maintained as described previously [14]. All mice were allowed access to water and food ad libitum and were maintained at a constant temperature (21 ± 3°C) and humidity (50 ± 10%) under a 12 h light/dark cycle (light on from 07:00–19:00). In this experiment, the hSOD1^G93A^ Tg mice were divided into two groups: Tg + Saline (S), *n* = 12, and Tg + *Scolopendra subspinipes mutilans* (SSM), *n* = 12. A total of 24 animals were used. All animals were handled in accordance with the animal care guidelines of the United States National Institute of Health and approved by the Institutional Animal Care and Use Committees of of the Korea Institute of Oriental Medicine.

### Scolopendra subspinipes mutilans (SSM) treatment

SSM was purchased from the Korean Pharmacupuncture Institute (Seoul, Korea). According to human acupoint landmarks and a mouse anatomical reference guide [15], the ST36 acupoint (Zusanli) is located 5 mm below and lateral to the anterior tubercle of the tibia. In previous studies, we demonstrated that the ST36 acupoint mediates anti-neuroinflammatory effects in the brain and spinal cord of hSOD1^G93A^ transgenic mice [14,16]. SSM (2.5 μℓ/g) was bilaterally injected (subcutaneously) at ST36 three times per week for two weeks, beginning when the mice were 98 days of age. The mice were then killed at 113 days of age. Control animals (hSOD1^G93A^) were bilaterally injected (subcutaneously) with an equal volume of saline at the ST36 acupoint.

### Tissue preparation

At 113 days of age, all mice were anesthetized with an intraperitoneal injection of pentobarbital and perfused with phosphate-buffered saline (PBS). For immunohistochemistry and immunofluorescence staining, the spinal cord tissue was removed and fixed in 4% paraformaldehyde for three days at 4°C prior to embedding. Briefly, the spinal cord was embedded in paraffin, and the prepared tissues were transverse sectioned (5 μm thick) and mounted on glass slides. Before staining, the sections were de-paraffinized in xylenes and rehydrated in a graded alcohol series, followed by dH_2_O.

### Nissl and Fluoro-Jade B (FJB) staining

Nissl staining was performed to evaluate general neuronal morphology and to demonstrate the loss of Nissl substance [17-19]; it was carried out as previously described [20]. Briefly, following de-paraffinization, the sections were allowed to dry in an oven and then stained with 0.1% cresyl violet, dehydrated through a graded alcohol series (70%, 80%, 90%, and 100%), placed in xylenes, and covered with a coverslip after the addition of histomount media.

FJB staining using a novel fluorescent marker with a high affinity for neuronal cells was conducted as previously described [21-23]. Briefly, de-paraffinized tissue was transferred to a solution of 0.1% potassium permanganate for 30 min, then rinsed with dH_2_O and transferred to a 0.05% FJB staining solution for 1 h in a darkroom. After washing, the sections were placed on a slide warmer (approximately 37°C for 30 min) and subsequently examined using a fluorescence microscope (Olympus Microscope System BX51; Olympus, Tokyo, Japan).

For the quantification of Nissl and FJB staining, we counted two sides of the anterior horn on every third section between the L4 and L5 levels of spinal cord. Cells were counted by a single person who was blinded to the identity of the treatment groups using the NIH program Image J (version 1.46j). The following criteria were used: (1) neurons located in the anterior horn ventral to the line tangential to the ventral tip of the central canal, (2) neurons with a maximum diameter of 20 μm or more, and (3) neurons with a distinct nucleolus [18,24,25].

### Immunohistochemistry and immunofluorescence staining

Following de-paraffinization, the slides were treated with 3% H_2_O_2_ to inactivate endogenous peroxidases and then blocked in 5% BSA in 0.01% PBS-Triton X-100 at room temperature. The sections were then incubated with the primary antibodies Iba-1 and GFAP overnight. The next day, the sections were washed with PBS and incubated in a 1:1,000 dilution of the primary matched-secondary antibody for 2 h. For visualization, an ABC kit and a DAB peroxidase substrate kit were used. Finally, the tissue sections were counterstained with cresyl violet, a Nissl stain, and coverslipped. Immunostained spinal cord sections were observed with a light microscope.

For immunofluorescence analysis, similar to the immunohistochemistry staining, sections were blocked in 5% BSA, followed by overnight incubation with a MAP2 primary antibody in 2% BSA. After three washes, the sections were incubated with a secondary antibody conjugated to FITC (1:1,000 dilution) for 2 h in the dark at room temperature and then mounted on glass slides using mounting medium with DAPI for fluorescence analysis. The slides were examined using a fluorescence microscope equipped with a filter cube designed for the visualization of FITC with green excitation light (450–490 nm) and a barrier filter.

For the quantification of immunofluorescence, the intensity of MAP2-positive cells were determined using Image J software. In each hSOD1^G93A^ mouse, all of the stained cells found within three spinal cord sections were counted. In each group, staining was performed in samples from six mice.

### Western blotting

Western blotting was conducted as previously described [26]. When the mice reached 113 days of age, their spinal cords and brainstems were dissected and homogenized in RIPA buffer (50 mM Tris–HCl, pH 7.4, 1% NP-40, 0.1% SDS, and 150 mM NaCl) containing a protease inhibitor cocktail. Following homogenization, a 20-μg sample of protein was quantified via the BCA assay. The samples were denatured with sodium dodecyl sulfate sampling buffer and then separated through SDS-PAGE, followed by transfer to a PVDF membrane. For the detection of target proteins, the membranes were blocked with 5% non-fat milk in TBS (50 mM Tris–HCl, pH 7.6, 150 mM NaCl) and subsequently incubated overnight with various primary antibodies: anti-tubulin, anti-iba-1, anti-GFAP, anti-CD14, anti-HO1, or anti-humanSOD1. The blots were next probed with peroxidase-conjugated secondary antibodies (Santa Cruz Biotechnology, CA, USA) and visualized using enhanced chemiluminescence reagents (Amersham Pharmacia, NJ, USA). Protein bands were detected with the Fusion SL4-imaging system (Fusion, Eberhardzell, Germany). Quantification of the immunoblotting bands was conducted with the NIH program Image J.

### Statistical analysis

All data were analyzed using GraphPad Prism 5.0 (GraphPad Software, CA, USA) and are presented as the mean ± SEM, where indicated. The results of immunohistochemistry and Western blot were analyzed using an unpaired *t*-test to compare the significance of the differences between the SSM and saline treatment at the ST36 acupoint in the ALS SOD1 transgenic mice. Statistical significance was set at *P* < 0.05 level.

## Results

### SSM treatment attenuates the loss of motor neurons in the spinal cord of symptomatic hSOD1^G93A^ transgenic mice

**Figure 1:**
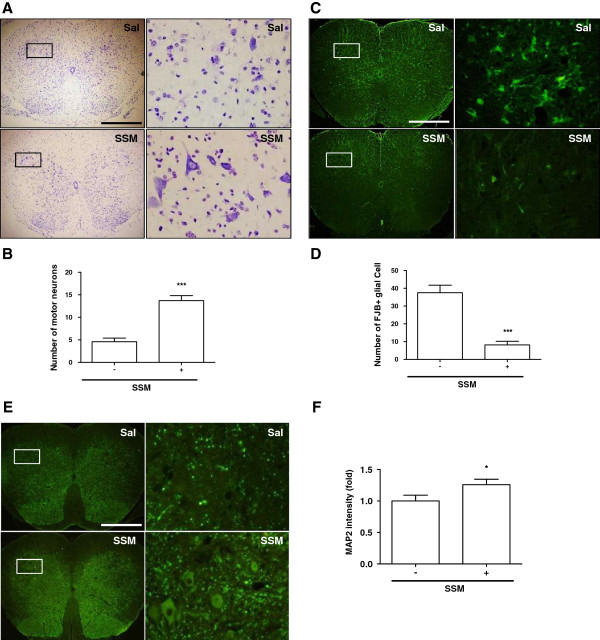
**SSM treatment increases motor neuron survival in hSOD1**^**G93A **^**mice.** SSM (2.5 μℓ/g) was administered bilaterally at acupoint ST36 three times per week for two weeks. Photomicrographs of Nissl **(A)** and FJB **(C)** staining of the lumbar spinal cord. Each right-hand column depicts a magnified image of the rectangular region of the corresponding image in the left column. The numbers of viable motor neurons **(B)** and degenerating FJB-positive glial cells **(D)** were counted, as described in the magnified spinal cord column. **(E)** Photomicrographs of MAP2 staining of the lumbar spinal cord. Each right-hand column depicts a magnified image of the rectangular region of the corresponding image in the left column. **(F)** Quantitative analysis of MAP2-positive cells each magnified column. Control (Sal) animals were bilaterally injected with an equivalent volume of saline at the ST36 acupoint. The data are presented as the means ± SEM (*N* = 6 animals/genotype). Statistical significance was assessed via *t*-test. ***P < 0.001 compared to the saline-treated group. Magnification: 100×. Bar = 500 μm. Sal: saline-treated hSOD1^G93A^ mice, SSM: Scolopendra subspinipes mutilans (SSM)-treated hSOD1^G93A^ mice.

To determine whether SSM treatment affects neuronal loss in the spinal cord of symptomatic hSOD1^G93A^ transgenic mice, we performed a histochemical assessment of lumbar spinal cord sections in SSM- and saline-treated mice. As shown in Figure 1A-B, we demonstrated via Nissl staining that the administration of SSM attenuated motor neuron loss in the ventral horn of L4-L5 segments of the spinal cord by 3.4-fold. To confirm the reduction of motor neuron loss induced by SSM treatment, we quantified the number of degenerated neurons through FB-J staining. As shown in Figure 1C-D, compared to age-matched control mice, the number of degenerated neurons in the ventral horn of the spinal cord was reduced by 4.6-fold in SSM-treated hSOD1^G93A^ transgenic mice. In addition, using an MAP2 antibody, we observed that neuronal cells were increased by 1.2-fold in the spinal cord of SSM-treated hSOD1^G93A^ mice compared to the saline-treated control mice (Figure 1E-F).

### SSM treatment reduces the numbers of microglial cells and astrocytes in the brainstems and spinal cords of symptomatic hSOD1^G93A^ transgenic mice

**Figure 2:**
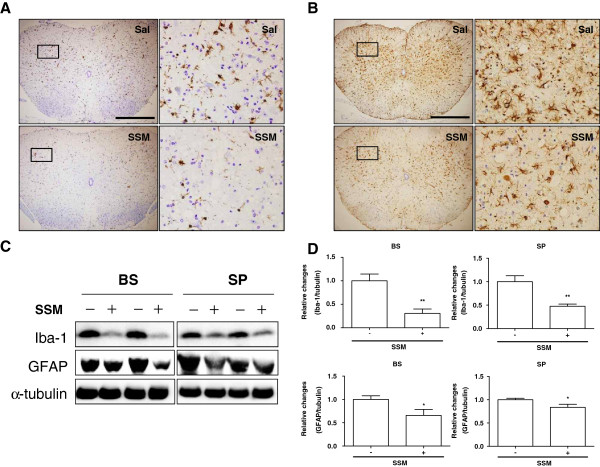
**SSM treatment reduces the expression of Iba-1 and GFAP in 113-day-old hSOD1**^**G93A **^**mice.** Representative photomicrographs of Iba-1 **(A)** and GFAP **(B)** staining in the lumbar spinal cord. Each column on the right depicts a magnified image of the rectangular region of the corresponding image in the left column. **(C)** Representative Western blot showing the activation of microglia using an Iba-1 antibody and the detection of astrocytes with a GFAP antibody in the brainstems and spinal cords of hSOD1^G93A^ mice. **(D)** Quantitative analysis of the levels of Iba1/tubulin and GFAP/tubulin, respectively. The data are presented as the means ± SEM (*N* = 6 animals/genotype). Statistical significance was assessed via *t*-test. **P < 0.01 compared to the saline-treated group. SSM: *Scolopendra subspinipes mutilans,* BS: brainstem, SP: spinal cord.

Neuroinflammation caused by activated microglial cells and astrocytes is the hallmark pathological feature of ALS in animals and patients [5-7,27,28]. To investigate the effects of SSM treatment on microglial cells and astrocytes in the spinal cords and brainstems of symptomatic hSOD1^G93A^ transgenic mice, we examined the expression of Iba-1 to visualize microglial cells and the expression of GFAP to visualize astrocytes in the brainstems and spinal cords of SSM-treated hSOD1^G93A^ transgenic mice and age-matched control mice. As shown in Figure 2A, SSM administration markedly reduced the massive activation of microglial cells (Iba-1-positive cells) and astrocytes (GFAP-stained cells) observed in the ventral horn of the lumbar spinal cord. In addition, biochemical analysis confirmed that the expression of Iba-1 was significantly reduced by 3.3- and 2.1-fold in the brainstem and spinal cord, respectively, compared to control mice (Figure 2C-D). Through Western blotting (Figure 2C- D), we found that, compared to age-matched control mice, the level of GFAP expression was decreased by 1.5- and 1.2-fold in the brainstems and spinal cords, respectively, of SSM-treated hSOD1^G93A^ transgenic mice.

### SSM treatment decreases inflammation and oxidative stress in the brainstems and spinal cords of symptomatic hSOD1^G93A^ transgenic mice

**Figure 3:**
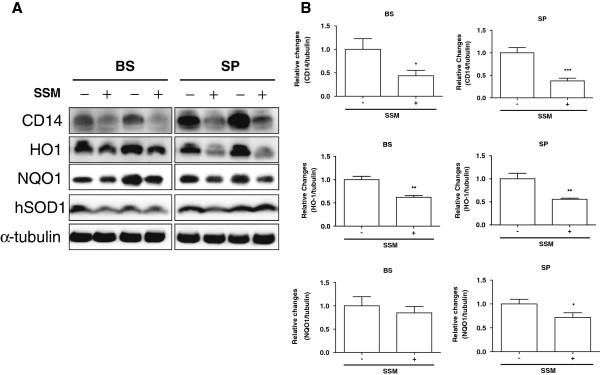
**SSM treatment regulates the expression of CD14, HO1, and NQO1 in the brainstems and spinal cords of hSOD1**^**G93A **^**mice. (A)** Representative Western blot showing the expression of CD14, HO1, and NQO1 in the brainstem and spinal cord following the administration of SSM. **(B)** Quantification of the level of CD14/tubulin, HO1/tubulin, and NQO1/tubulin. The data are presented as the means ± SEM (*N* = 4 animals/genotype). Statistical significance was assessed via *t*-test. ***P < 0.001, **P < 0.01, and *P < 0.05 compared to the non-treated group. BS: brainstem, SP: spinal cord.

To confirm the anti-neuroinflammatory effects of SSM treatment in the brainstems and spinal cords of hSOD1^G93A^ transgenic mice, we examined the expression of CD14, a component of the innate immune system. As shown in Figure 3A, the expression of CD14 was reduced by 2.3- and 2.7-fold in the brainstems and spinal cords, respectively, of SSM-treated hSOD1^G93A^ mice. Furthermore, we found that the administration of SSM reduced the expression of the HO1 protein by 1.6- and 1.8-fold in the brainstem and spinal cord, respectively. To confirm the effect of SSM on oxidative stress, we examined the expression of NAD(P)H dehydrogenase (quinone 1) (NQO1) in the brainstem and spinal cord of hSOD1^G93A^ transgenic mice. SSM treatment reduced the expression of NQO1 by 1.4-fold in the spinal cord of symptomatic hSOD1^G93A^ transgenic mice.

## Discussion

In this study, we provided evidence that the administration an SSM extract attenuates neuroinflammation in symptomatic hSOD1^G93A^ transgenic mice. SSM treatment significantly reduced the numbers of microglial cells and astrocytes and the loss of degenerated neurons in the spinal cords of hSOD1^G93A^ transgenic mice.

ALS is an adult-onset motor neuron disease caused by mutations in the genes encoding Cu/Zn superoxide dismutase (SOD1), alsin, senataxin, (FUS), vesicle-associated membrane protein-associated protein B (VAPB), TAR DNA-binding protein (TARDBP), and dynactin 1 (DCTN1) [3]. Specifically, transgenic mice bearing a mutant form of human SOD1 have been used to determine the pathological mechanisms of ALS and to identify effective therapies for ALS patients. Familial ALS (fALS), caused by inherited gene mutations, accounts for approximately 5-10% of all ALS cases, whereas sporadic ALS (sALS), which does not display a genetic component, accounts for the majority of ALS cases. Although several pathological mechanisms involved in ALS, such as neuroinflammation, oxidative stress, mitochondrial dysfunction, and glutamate excitotoxicity, have been reported [3], it remains unclear how to develop therapies for the treatment of both fALS and sALS patients. SSM contains many types of proteins, 5-hydroxytryptamine, histamines, lipids, polysaccharides, and various enzymes (e.g., proteinases and estrases) and has been used to treat various diseases, including cancer, stroke, and epilepsy in Chinese and Korean traditional medicine [8,9,29,30]. SSM treatment has been reported to have many biochemical and physiological effects [8,31]. Especially, Ren et al. [32] have shown the antiinflammatory effects of SSM in Alzheimer’s disease. In the present study, we showed that SSM administration attenuated the loss of motor neurons in the ventral horn of the spinal cord in symptomatic hSOD1^G93A^ transgenic mice (Figure 1). In addition, we found that SSM treatment significantly reduced the degeneration of neuronal cells in the lumbar spinal cord (Figure 1B), suggesting that the administration of SSM could be a useful therapy for neurodegenerative diseases.

The nervous system consists of neurons and glial cells, including astrocytes and microglia. Microglia are the resident immune cells of the nervous system and provide protection against infection. However, microglial cells become activated in neurodegenerative disorders and are involved in neuroinflammation, leading to neuronal cell death. Astrocytes contribute to homeostasis by providing energy and substances required for neurotransmission in the brain. In addition, glia play a role in promoting neuronal survival by forming a network between neurons and astrocytes. However, activated astrocytes react to various pathological events by releasing toxic factors and developing excitotoxicity, which kills motor neurons in individuals with ALS [33,34]. Neurodegenerative disorders such as AD, PD, and ALS are caused by neuroinflammation. In both ALS patients and animal models of ALS, microglia and astrocytes are highly activated when motor neuron loss occurs [35,36]. Yamanaka et al. have demonstrated that expression of the hSOD1 mutation in astrocytes induces motor neuron death and increases the severity of diseases involving microglial activation [36]. Therefore, we examined whether SSM treatment affects neuroinflammation in the brainstems and spinal cords of symptomatic ALS animals. First, we confirmed the loss of neurons through Nissl and FBJ staining and then measured neuronal cells via MAP2 labeling in the spinal cords of ALS mice (Figure 1). SSM treatment reduced neuronal death in the ventral horn of the lumbar spinal cord in hSOD1^G93A^ transgenic mice (Figure 1). In addition, we found that SSM administration decreased the activation of microglia and astrocytes in the brainstems and spinal cords of mutant hSOD1 mice (Figure 2). Furthermore, we confirmed that the level of inflammation-induced CD14 in the brainstem and spinal cord of mutant hSOD1 mice was markedly reduced by the administration of SSM (Figure 3). Although the exact mechanisms underlying the effects of SSM treatment in ALS animals have yet to be elucidated, this study suggests that SSM treatment could help to attenuate the neuroinflammation associated with ALS.

The exploitation of antiinflammatory agents is a widely studied topic in neurodegenerative disease research. The results of the present study contribute to clarifying the mechanisms underlying the antiinflammatory effects of SSM in relation to the effective treatment of neurodegenerative diseases. However, additional studies are necessary to identify the bioactive components of this centipede that are responsible for its antiinflammatory effects. In addition, the effects of SSM on motor function and the survival of hSOD1^G93A^ transgenic mice should be examined in future studies.

Oxidative mechanisms have been implicated in a number of pathological states affecting both the central and peripheral nervous systems. Oxidative stress has also been implicated in the pathology of some neurodegenerative disorders. Several studies have revealed increases in oxidative stress, including elevated carbonyl levels, 3-nitrotyrosine levels, and lipid oxidation, in ALS cases [37-39]. Oxidative stress mediates other pathological mechanisms, such as glutamate excitotoxicity, ROS production, and mSOD1 aggregation in ALS [3]. An inducible isoform of hemeoxygenase 1 (HO1) and NAD(P)H dehydrogenase (quinone 1) (NQO1) are expressed as a response to stress, including oxidative stress, as are cytokines [40-42]. To determine the effect of SSM on oxidative stress in ALS animals, we examined the expression level of HO1 and NQO1 in the brainstems and spinal cords of saline- and SSM-treated hSOD1^G93A^ mice. Compared to age-matched control mice, we found that SSM treatment significantly reduced the expression of HO1 and NQO1 in the spinal cords of symptomatic hSOD1^G93A^ transgenic mice (Figure 3), suggesting that the antiinflammatory effect of SSM may occur through the reduction of oxidative stress in the brainstems and spinal cords of hSOD1^G93A^ transgenic mice. Therefore, to develop effective ALS therapies in the future, it will be necessary to determine whether SSM can penetrate the CNS.

## Conclusions

SSM treatment at acupoint ST36 reduced the neuroinflammation and HO1 and NQO1 expression induced by oxidative stress in the ventral horn of the spinal cord of symptomatic hSOD1^G93A^ transgenic mice. Furthermore, motor neuron loss in the spinal cord was attenuated by SSM administration. Based on these findings, we suggest that SSM treatment could be useful as an anti-neuroinflammatory therapy for neurodegenerative diseases.

## Abbreviations

ALS: Amyotrophic lateral sclerosis; SSM: *Scolopendra subspinipes mutilans*; CAM: Complementary and alternative medicine; fALS: Familial ALS; SOD1: Superoxide dismutase; FUS: Fused in sarcoma; VAPB: Vesicle-associated membrane protein-associated protein B; TARDBP: TAR DNA-binding protein; DCTN1: Dynactin 1; sALS: Sporadic ALS; hSOD1: Human SOD1; AD: Alzheimer’s disease; PD: Parkinson’s disease; FB-J: Fluoro-Jade B; NQO1: NAD(P)H dehydrogenase (quinone 1).

## Competing interests

The authors declare that they have no competing interests.

## Authors’ contributions

EJY designed the experiments and analyzed the data as well as wrote the manuscript. MDC executed all the experiments, and SMC, BKS, and IS discussed the manuscript. SCK discussed the data and edited the manuscript. All authors have read and approved the final manuscript.
